# Assessing Visual Exploratory Activity of Athletes in Virtual Reality Using Head Motion Characteristics

**DOI:** 10.3390/s21113728

**Published:** 2021-05-27

**Authors:** Markus Wirth, Sebastian Kohl, Stefan Gradl, Rosanna Farlock, Daniel Roth, Bjoern M. Eskofier

**Affiliations:** 1 Machine Learning and Data Analytics Lab, Department of Artificial Intelligence in Biomedical Engineering (AIBE), Friedrich-Alexander-Universität Erlangen-Nürnberg (FAU), Carl-Thiersch-Straße 2b, 91052 Erlangen, Germany; sebkohl@web.de (S.K.); stefan.gradl@fau.de (S.G.); bjoern.eskofier@fau.de (B.M.E.); 2 Adidas AG, Adi-Dassler-Straße 1, 91074 Herzogenaurach, Germany; Rosanna.Farlock@adidas.com; 3 Human-Centered Computing and Extended Reality, Department of Artificial Intelligence in Biomedical Engineering (AIBE), Friedrich-Alexander-Universtität Erlangen-Nürnberg (FAU), Henkestr. 91, 91052 Erlangen, Germany; d.roth@fau.de

**Keywords:** virtual reality, visual exploratory activity, perceptual–cognitive skills, head turn activity

## Abstract

Maximizing performance success in sports is about continuous learning and adaptation processes. Aside from physiological, technical and emotional performance factors, previous research focused on perceptual skills, revealing their importance for decision-making. This includes deriving relevant environmental information as a result of eye, head and body movement interaction. However, to evaluate visual exploratory activity (VEA), generally utilized laboratory settings have restrictions that disregard the representativeness of assessment environments and/or decouple coherent cognitive and motor tasks. In vivo studies, however, are costly and hard to reproduce. Furthermore, the application of elaborate methods like eye tracking are cumbersome to implement and necessitate expert knowledge to interpret results correctly. In this paper, we introduce a virtual reality-based reproducible assessment method allowing the evaluation of VEA. To give insights into perceptual-cognitive processes, an easily interpretable head movement-based metric, quantifying VEA of athletes, is investigated. Our results align with comparable in vivo experiments and consequently extend them by showing the validity of the implemented approach as well as the use of virtual reality to determine characteristics among different skill levels. The findings imply that the developed method could provide accurate assessments while improving the control, validity and interpretability, which in turn informs future research and developments.

## 1. Introduction

Physical skills and abilities of athletes have been examined and improved for decades applying real-time [1,2] or post-game [3] analysis. Consequently, sophisticated methods are established to assess and train relevant physical properties of athletes depending on the type of sports [4,5,6]. While physical and technical aspects are decisive to maximize performance, gaining superior skills encompasses performance aspects beyond solely individual and isolated movement execution, especially in team sports.

In fast-paced team sports, like soccer, athletes have to face an environment that is highly unpredictable and unstable. A large field including 22 players showing constant movement makes the game dynamic and complex, resulting in almost infinite possibilities to act and react. Athletes are forced to focus their attention on relevant information sources for efficient decision-making and anticipation processes. Consequently, with pro athletes showing superior skills in these areas, knowing more about them is beneficial for various areas like talent identification and performance improvement [7]. Sports science has identified relevant cognitive and perceptual characteristics, like the ability to recognize task-related patterns [8,9,10] and VEA [11,12,13] that lead to superior on-pitch performance.

Visual exploration enables athletes to gain environmental information. In this sense, exploratory actions entail eye, head and body movements supporting the identification of opportunities for successful acting within a 360° environment in soccer. To investigate VEA, a broad variety of studies focuses on gaze behavior of athletes using eye tracking technology [14]. These studies derive recommendations about context-specific and efficient visual exploratory behavior based on multiple gaze measures, including the number and duration of fixations, saccades and viewing time [15]. However, the application of eye tracking is still cumbersome to implement and necessitate expert knowledge to interpret results correctly [14].

Taking an easily applicable approach, the potential of head movement metrics within VEA is not holistically explored. First research in this area was able to identify parameters like the frequency or excursion of head turns to be relevant [16]. Nevertheless, these studies often lack to provide realistic scenarios, displaying simple video-based stimuli, or show a low reproducibility (e.g., on-pitch evaluations). To further explore the potential of head turn movements and hence evaluate the importance for performance analysis, more sophisticated approaches are needed, to provide a realistic and controlled assessment environment.

In this regard, technology had and continues to have a major impact on sports. For example by allowing a deeper understanding of athletes’ performance in diverse sport situations and by providing decision support for coaches and athletes. Therefore, sensor- and display-based methods were developed and employed to establish meaningful objective performance measures [17,18,19,20].

Compared to traditional media formats, VR can allow for a greater degree of presence and hence enable training experiences closer to a realistic game context [21]. It can further function as an amplifier for interest, motivation, and engagement of users regarding their task [22,23]. The application of VR allows for the creation of an interactive sport assessment system in a sense that user actions can influence the behavior of the digital content using controller modalities and user behavior. Further, while the sensors embedded in an HMD are primarily used for determining the camera pose, they can simultaneously function as valuable data source to derive movement-based assessment measures like HTF or HTE [24,25]. Since every object and action within a VE can be predefined, precise monitoring at any point in time is possible which in turn allows comparable evaluations of performance metrics [26].

Even though it could be shown that inertial sensors can be used for assessing athletes exploratory behavior in different sport situations [24,25], little research has been conducted using HMD integrated sensors to derive performance measures [27,28]. Specifically, to the best of our knowledge, no work investigates the assessment of VEA based on head movement characteristics under the condition of realistic dynamic VR-based soccer scenarios.

### Contributions

In this paper, we contribute the design and implementation of a dynamic virtual soccer environment, consisting of multiple virtual plays that are created using real-world tracking data. We demonstrate the use of the simulation to assess the head motion behavior of soccer players while performing a recognition and recall task. We propose an assessment strategy and pipeline consisting of six different head turn measures based on head velocity data gathered from inertial sensors integrated into an HMD. Additionally, we utilized the gained data to investigate the focus area of athletes by determining the cFOV time on relevant objects (i.e., ball, opponents and teammates). In a user study including 28 soccer players of two different skill levels, we investigated the plausibility of the developed analysis pipeline. We further assessed the quality of the VE by measuring the experienced presence and the user experience of participants.

Results show significant differences between skill levels for the head turn measures HTC, HTE, HTF, as well as HTVel and its variability. Significant differences could also be observed for the duration of centered objects. Further, we found a significantly high correlation between the HTE and the experienced presence. The findings in this work demonstrate the validity of the investigated measures and VR as a medium for assessing the exploratory performance of team athletes. Resulting implications are important for manifold team sports assessment simulations and guide further research.

## 2. Background and Related Work

The human process of perception comprises interpretation and understanding of geometrical shapes or objects, registered by the visual system [29]. Regarding the information process theory of Haber and Hershenson, interpretation is a collaboration of the central nervous system and working long-term memory [30]. For interpretation and organizing, not only the efficient transfer of environmental stimuli is decisive for sense-making, but also previous experiences stored in the long-term memory. Memory is based on human experiences and further enables to build domain-specific structures that result in superior performance [31,32]. Therefore, the ability of efficient visual exploration is an essential component for human decision-making and action execution.

VEA in sports encompasses all movements of the eye, head and body in regards to perceiving relevant environmental stimuli prior to dedicated events and actions [33,34,35]. In team sports, it is decisive for athletes to register all situation relevant information which requires active looking behavior and hence full task engagement. This implies a constant alignment of orientation and attention regarding rapidly changing sources of information such as the position of opponents and/or teammates. Superior decision-making further requires efficient selection, processing and filtering of relevant information [36]. To understand the effect of certain perception and search strategies on decision-making and action execution, previous research exists using laboratory and on-pitch evaluations.

To investigate search behavior and perception of athletes most prevalent eye tracking is used in previous studies for the quantification of performance. Therefore, different dependent variables like the mean, location, duration and total number of fixations as well as amplitude, frequency, latency or velocity of saccades are used. In a review conducted by McGuckian et al., the number of fixations and fixation duration could be identified as the most dominant measures to be applied for analysis [14]. Even though yielding great potential, results of previous research are heterogeneous towards their meaning and usability for performance analysis in soccer. When comparing experienced and inexperienced players some literature concludes [12,32,37], that the frequency of fixations is higher for more experienced players. In a study conducted by Roca et al., skilled and less skilled players experienced life-size 11 vs. 11 soccer situations in which the ball was located in the defensive or offensive half of the pitch. Overall, higher-skilled players showed more accurate anticipation and decision-making skills accompanied by a significantly higher mean number of fixation and shorter fixation duration [38].

In contrast, there exists research that found fewer fixations for experienced or more skilled soccer players [9,39,40]. Cañal-Bruland et al. conducted a study, presenting overview images of offensive and defensive game situations to a total number of 56 participants. Their study could show that skilled soccer players performed significantly fewer fixations of longer duration for decision-making compared to their counterparts. Based on the results they concluded that skilled players extract more information from a single glance [39].

In a recent study conducted by Aksum et al., the search behavior of five players in the Norwegian premier league in an 11 vs. 11 game was investigated [41]. They could observe higher fixation rates of players in case more information sources became available. Further, a shorter fixation duration could be observed in real-world game situations compared to laboratory results. The authors claim that simple laboratory studies using media like images or videos on screens may not be sufficient to capture soccer players experience under match-play conditions.

Further studies investigated the head activity of athletes to draw conclusions on exploratory activity. Relevant measures in these studies are the duration, excursion and frequency of a head turn. The majority of these studies uses a manual evaluation process annotating relevant actions supported by video footage to analyze the behavior of participants [42,43,44]. Eldridge et al. examined the effect of VEA of midfield soccer players in relation to their actions using post-event video analysis. Within their study, VEA refers to players’ body and/or head movement away from the ball with the goal of searching for teammates, opponents or events in the environment prior to receiving the ball. Study results could show that players experienced significantly less pressure when performing more VEA before receiving the ball and made more passes into the attacking half. Overall, they claim to present evidence that more visual activity can influence tactical and technical aspects of performance. However, even though video-based manual annotation approaches can be applied for determining the visual activity of athletes, they show disadvantages due to labor intensity and are prone to human errors [42].

Other research suggests the application of IMUs or MEMS to determine the VEA of athletes. Using a head-worn sensor-based method, Chalkley et al. defined a head turn as a distinct movement along the longitudinal axis that exceeds an angular velocity of 125° s^−1^ [24,45]. To validate their approach one participant wearing an IMU instrumented headband was placed in the center of five pre-defined labeled targets. During the experiment, the participant was asked to perform three sequences of 24 random selected trials looking at different targets. For gold standard validation, a total of 72 head turns were counted manually using video recordings. The result could be confirmed by the applied algorithm, detecting the same amount of head turns. Several follow up studies adapted this approach to investigate the effect of VEA in relation to affordance [25], on-ball performance [46], playing role [47] and passing performance [16]. To explore the latter, McGuckian et al. conducted a study in which fourteen high-skilled U13 and U23 soccer players conducted a 32-trial sequence within the Footbonaut. The Footbonaut (CGoal GmbH, Berlin, Germany) is a system that includes eight ball-dispensers and 64 square target gates. Balls can be dispensed at varying speeds, angles and spin to the athlete centered in the middle. To display stimuli (i.e., target the ball should be kicked to), gates are visually highlighted. During the study the head turn count, frequency and excursion were measured during and before ball possession. Results could show that a faster performance can be associated with a higher number of head turns before ball possession and a lower number of head turns during ball possession [16].

In summary, previous research investigating the implications of VEA mainly employed methods that are based on simple displaying procedures like 2D video clips, images or require labor and/or cost-intensive systems like the Footbonaut. However, due to high immersion and presence, VR has great potential for the assessment of behavior. Especially when it comes to identifying and developing new objective metrics to quantify athletes’ performance, only few research is conducted. Addressing this research gap is crucial since it provides the possibility of getting deeper insights into the behavior of athletes while being able to provide a realistic 360° reproducible environment based on stereoscopic vision.

For different team sports there exists previous work investigating the feasibility of VR for assessing the decision-making process of team sports athletes [19,23,48]. In a study conducted by Wirth et al. they used 360° video footage displayed via an HMD to investigate PCS of soccer players [19]. Therefore, a variety of pre-recorded 5 vs. 5 in vivo game scenes were experienced by amateur and expert players. Results revealed a shorter reaction time for expert players. Further, the habituation-time of amateur players was found to be significantly longer compared to their counterparts.

Previous research work also investigated the applicability of VR for improving the decision-making process of quarterbacks [23,49]. In a study conducted by Huang et al. an HMD was used to examine the effect of a VR-based training protocol on situational awareness of American Football quarterbacks. Different match-game situations were simulated in the virtual environment and trained over a period of three days. Results could show an increase of 30% in pre-snap reads and in-game decision-making [23].

Other research work addressed anticipation skills of athletes [50,51,52]. McLeod et al. examined different ball heading strategies of soccer players using VR [50]. Therefore, they altered the ball’s trajectory in mid-flight from a normal quasi-parabolic path to a linear one. Results suggest that players use a combination of expectation and memory to determine the rate of change that enables them to follow a ball during flight. Comparable to this study, Hoinville et al. employed the Magnus effect to create a spinning ball simulation in a VE [51]. Experienced and novice soccer players were asked to head during a free kick with and without sidespin. The qualitative results of the study showed that the interception performance is dependent on the ball spinning direction and arrival position for both groups.

Additionally, the anticipation of rugby players was evaluated by Bideau et al. using VR [53]. In their experiment, they tested the ability of novice and expert rugby players to detect deceptive movements in attacking player’s run. Therefore, participants had to define the final running direction of the virtual agents under different occlusion conditions. Results of the study could reveal a significantly higher positive decision rate for experts compared to novices. Experts also showed quicker anticipation and decision-making processes. Most recent research conducted by Ferrer et al. examined the read-the-game skills of soccer players in VR [27,54]. According to the authors this ability encompasses VEA, necessary for accurate in-game decision-making [55]. For measuring VEA the long exploratory activity and 180° exploratory activity was determined dependent on participants’ head orientation while experiencing a VR-based soccer scene with a duration of seven seconds. Results show that there is a significant difference in VEA between beginners and amateur soccer players.

In summary, whereas there is research conducted investigating the performance of athletes using VR, these evaluations are mainly based on simple measures (e.g., successful actions or reaction-time) and hence do not characterize athletes’ performance in more detail. Precisely, to the best of our knowledge, there is no work that examined head motion characteristics for defining VEA of athletes in dynamic game situations using VR. To address this research gap, we developed and evaluated a new VR-based method, including 18 dynamic soccer plays, that uses different head turn-based measures, namely the head turn frequency, the duration and the excursion as well as average head turn velocity and its variability to characterize VEA of athletes. Further, athletes’ FOA is evaluated by determining the duration of relevant objects within their cFOV. The developed method gives deeper insights into athletes’ performance and consequently can be used to optimize individualized training as well as talent development and scouting processes.

## 3. Virtual Performance Assessment Environment

To create the virtual soccer environment, real-world soccer plays were mapped on agents within the VE including the ball. Therefore, nine 6 vs. 6 soccer plays were recorded using a LPS. The positioning data was pre-processed and mapped on soccer agents and a virtual ball. Also, a realistic stadium scene was created. To do so, existing assets from the Unity asset store (https://assetstore.unity.com/packages/3d/characters/soccer-players-stadiums-pack-105891, accessed on 25 May 2021) were utilized and modified, fulfilling the international football standards recommended by the International Federation of Association Football (FIFA) [56].

### 3.1. Data Acquisition and Play Design

**Figure 1:**
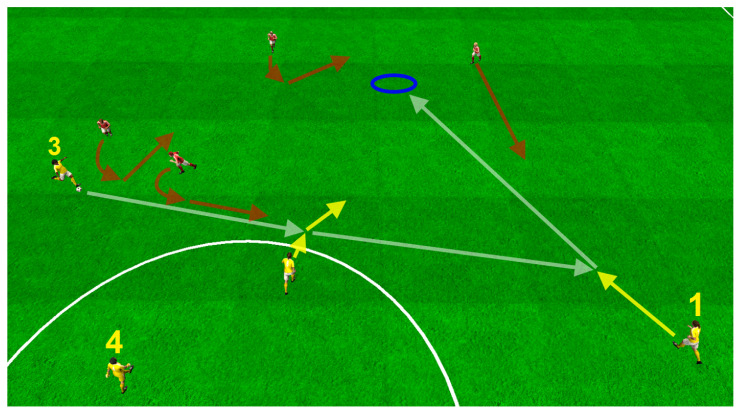
The illustration shows the sequence of an exemplary virtual play, including the movement paths of the individual players (yellow and red) and the ball (white). The blue circle outlines the participant’s position within the play.

Real-world recorded positioning data of 11 soccer players was used to design a realistic soccer simulation. These players performed a total of nine plays representing offensive and defensive game situations. To ensure a high level of plausibility, the structure and procedure of the plays were designed by sport experts. Each game situation consisted of two teams, including five teammates and six opponents. Every play ended with the user receiving a pass. The nine plays varied in the movement of the teammates and opponents. Furthermore, two user positions were defined. A central position closely oriented behind the striker was chosen for the offensive plays. For simulation of the defensive situations, the user was positioned close to the central defender. Figure 1 depicts an example of a resulting virtual play.

For data recording of these plays, we used a Kinexon LPS tracking system. The system consisted of 14 antennas placed around an outdoor soccer pitch. Data was collected at 20 Hz using UWB technology in a frequency range of 4.25–7.25 GHz.

To enable the data acquisition, soccer players were equipped with a sensor beacon on the back including a 9-axis IMU. Additionally, a beacon was attached to the inside of the ball. All players were briefed individually regarding their movement behavior to be performed. For each play, multiple data acquisitions were made. The most adequate dataset was selected by two sports science experts depending on the players’ behavior. Therefore, additional video footage from a bird’s eye view camera placed on a stand next to the pitch was used as a reference. For the nine different plays, 2D relative positioning data represented by x and y coordinates on the tracked soccer pitch were recorded as well as the velocity of the individual players and the ball measured in ms^−1^. To increase the number of and decrease the memorability of individual plays, the nine originally recorded plays were visually mirrored in the virtual simulation. This resulted in a total of 18 plays. Further, the appearance of the soccer agents was changed. The faces, skin colors, hairstyles, as well as jersey colors and numbers of opponents and teammates were randomly assigned for each play.

### 3.2. Animation of Soccer Agents

**Figure 2:**
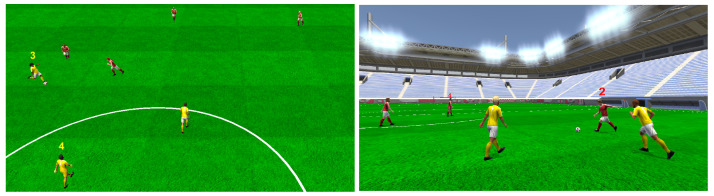
The left image shows an overview of an example soccer play, which was created using real-world tracking data. The right image depicts another example of a play within VR from the perspective of the user. The numbers above the virtual soccer agents’ heads represent the stimuli used for the recognition and recall task to be performed. Note that for the recall task, jersey colors were randomized.

To animate the virtual agents representing teammates and opponents within the simulation as well as the ball, pathways were defined based on the acquired real-world staged play data (Figure 2). The movement of the virtual objects along these paths was implemented without temporal deviations by calculating the position changes using linear interpolation. Meaning that the position was computed for every path piece between every path point pair as follows:

(1) $$f\left(t\right)=f_{0}+\frac{f_{1}-f_{0}}{t_{1}-t_{0}}\left(t-t_{0}\right),t\in \left[t_{0},t_{1}\right]$$

where *t* is the time and f(t) describes the interpolated position in the interval $\left[t_{0},t_{1}\right]$.

The moving direction was used to determine the orientation of agents. An agent’s body was centrally aligned in respect to the movement vector. To simulate appropriate locomotion of agents, four moving animations were applied. These animations varied in the behavior of the skeletal model including idle, jogging, running and sprinting. The animations were pre-recorded and part of the used assets package from the Unity asset store (https://assetstore.unity.com/packages/3d/characters/soccer-players-stadiums-pack-105891, accessed on 25 May 2021).

An animation for passing actions was needed to represent the real-world recording in VR. Therefore, the reference video footage was used to manually label the passing actions within the dataset. These labels were used as a trigger for a passing animation and orienting the soccer players towards the ball within the VE. The utilized animations were part of the previously named asset package. For providing a smooth transition between the individual animations, a transition time of 250 ms was determined empirically.

## 4. Evaluation

### 4.1. Study Design

To evaluate our proposed method we conducted a repeated-measures experiment including a recognition and recall task. The level of professionalism of the participants served as between factor. We compared between two skill levels of soccer players (inexperienced and experienced, see Section 4.4). The task was performed in a virtual soccer environment consisting of the 18 introduced virtual soccer plays. These plays were presented in a randomized order in each trial. Every participant performed two trials (i.e., 36 plays in total). Dependent variables were the head motion characteristics of participants as well as their recognition and recall performance. Further, the focus time of athletes on the three different object types ball, teammates and opponents was determined. The quality of the VE was evaluated by measuring user experience.

#### 4.1.1. Task Design

The applied recognition and recall task was implemented by using different visual stimuli. These stimuli consisted of a varying number of marked agents that had to be verbally named in numbers by the participant after experiencing a play. For marking, a label consisting of the respective jersey number and color of an agent was placed above its head (Figure 2).

The number of marked agents was randomly chosen for each play and ranged between one and five players. Even though this varied over the individual plays, it was equally balanced across all participants. To create a fair marking of agents, the relevance of all teammates for each play was ranked by a sports expert. This enabled a prioritized marking of agents and hence ensured the comparability of the experiment.

### 4.2. Hardware

For displaying the virtual soccer environment, the HTC Vive Pro HMD with a refresh rate of 90 Hz, equipped with a wireless adapter, was utilized. The HMD has a 110° FOV consisting of two displays with a resolution of 1440 × 1600 pixels. For rendering the simulations, a PC equipped with the AMD Ryzen Threadripper 1950X16-core processor (3.40 GHz), a 32 GB installed RAM, and the NVIDIA GeForce RTX 2080 Ti graphics card was used.

### 4.3. Measures

To calculate the head movement-based measures, the orientation of the HMD was recorded in 3D space for each frame. A Savitzky–Golay filter was applied for smoothing the data [57]. It performs a polynomial regression over a series of values to determine a smoothed value for each point. The first derivative with a window size of three was used to calculate the velocity.

#### 4.3.1. Head Turn Count and Frequency

The HTC describes the head turns during a specific time frame. A head turn in sports is defined as a fast rotation of the head around the vertical axis of the body, exceeding an angular velocity of 125° s^−1^. The HTF is the equivalent time-normalized parameter, given in turns per second [16,25].

#### 4.3.2. Head Turn Excursion and Duration

The excursion of a specific head turn is described by the HTE. It quantifies the absolute radian measure between the beginning and end of a detected head turn in degree. Likewise, the HTD describes the duration of a head turn [16,25].

#### 4.3.3. Head Turn Velocity and Variability

In this work we define the HTVel as the velocity around the vertical axis also referenced as yaw-velocity. The HTCoV is calculated using yaw-velocity. It is defined as the standard deviation in relation to the mean and therefore describes the variability of velocity. It is represented in percent. Concerning sports, we see the HTCoV as an indicator of an athlete’s volatility in head motion.

#### 4.3.4. Focus of Attention

For the FOA the cFOV is chosen as a reference to investigate the focus of an athlete in relation to different objects within the VE. To determine the focused object, the minima of the Euclidean distance between the rendered objects and the cFOV is used as a selection criterion for each frame. The FOA is measured in time (s) spent on predefined objects (i.e., ball, opponents and teammates).

#### 4.3.5. Recognition and Recall

The RRS reflects the capability of soccer players being able to recognize and recall the number of stimuli during a play (see Section 4.1.1). The RRS is a comparison between the actual and realized visual stimuli by the participant for a play. The measure was chosen to be sensible to under- and overestimation and hence can be described as:

(2) $$RRS=f\left(a\right)=1-\frac{\left|s-a\right|}{s}=\left\{\begin{array}{cc} f(a), & f(a)>0\\ 0, & f(a)\leq 0 \end{array}\right.$$

whereas *s* is the number of visual stimuli presented within the virtual environment and *a* is the number of stimuli recognized by the user.

#### 4.3.6. Presence

To determine the presence of participants within the virtual soccer environment, the PQ as recommended by Witmer and Singer [58] was used. This questionnaire consists of 25 items on a 7-point Likert scale. Each item includes semantic differentials. The factors of the questionnaire can be classified into control (CF), distraction (DF), realism (RF) und sensory (SF). Since no audio feedback was used during the experiment, the relevant questions regarding this factor were excluded.

#### 4.3.7. User Experience

For evaluating the user experience of participants, we used the UEQ [59]. It includes 26 items with a 7-point Likert scale of semantic differentials aggregated by the six factors attractiveness (ATT), perspicuity (PER), efficiency (EFF), dependability (DEP), stimulation (STI), and novelty (NOV). Further, a benchmark dataset ($BM_{UEQ}$) consisting of data from 18,483 people collected in 401 studies is provided. The $BM_{UEQ}$ allows categorizing ratings into five intervals namely— excellent, good, above average, below average, and bad [60].

### 4.4. Participants

A total of 28 soccer players (1 female, 27 male) with an average age of M=26 (SD=7) years participated in the study. 27 of the participants had no experience with VE before. All of the participants were active soccer players and had a mean soccer experience of M=17.5 (SD=6.8) years. The participants had an average height of M=179.5 (SD=7.5) centimeters. For analysis purposes the soccer players were divided into an inexperienced and experienced group. Whereas inexperienced athletes are defined playing in the two lowest German soccer leagues, the experienced group was defined as soccer players actively playing in the third-lowest league or higher. This resulted in a group size of 11 experienced and 17 inexperienced players.

### 4.5. Procedure

Participants were welcomed and gave informed consent to be aware of risks associated with the conducted study. Then they were asked to give demographic information by filling out a questionnaire. Next, the study procedure was explained and the HMD was calibrated by adjusting the lens-to-eye distance and lens distance. Five introduction plays were shown, to ensure that the participants were familiar with the virtual soccer environment and the task to be performed.

Following, each participant experienced the 18 unique plays in a randomized order during one trial. In total, two trials were performed. Within one play, some of the teammates were marked with labels above their heads, representing the number printed on their jersey in the respective jersey color. Before the beginning of a simulated play, participants were placed at one of the two pre-determined positions (see Section 3.1). From this position, they were able to observe the actions and soccer environment with a FOR of 360° in 3D space including depth perception while standing. As a final act of each play, a pass was played to the participants before the scene was paused and player agents as well as the ball were blanked out. Participants were then asked to give verbal feedback regarding the number of marked agents and the most promising action to be taken next. To give feedback regarding their performance, the labels and hence the number of agents was shown after an answer was given.

**Figure 3:**
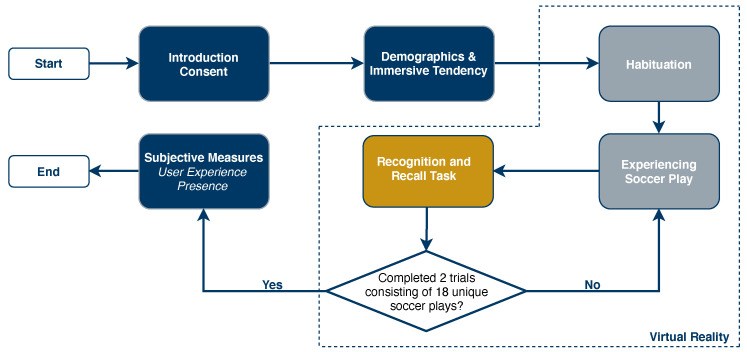
Study procedure.

For each play data on participants head movement was acquired before receiving the ball. After experiencing the two trials, participants were asked to rate the quality of the simulation by filling out the UEQ and PQ. Figure 3 shows an overview of the study procedure. Since the data recording took place before the COVID-19 pandemic, regular hygiene measures were taken for the study.

### 4.6. Analysis Strategy

For statistical analysis of significance, the two groups were compared applying a Welch’s two-sample t-test. To examine the linear correlation between individual measures, we calculated Pearson’s correlation coefficient. For all statistical measures a significance level α=.05 was determined.

## 5. Results

### 5.1. Head Turn Performance

**Figure 4:**
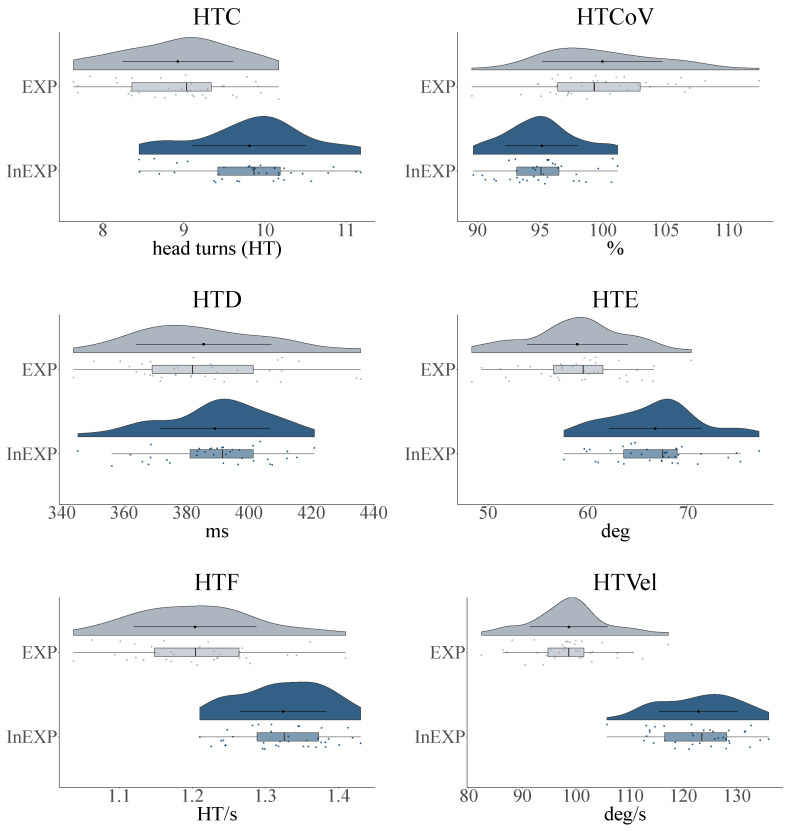
Illustration of the data distribution and probability density for inexperienced (InEXP) and experienced (EXP) soccer players of the individual head turn characteristics. Head turn count (HTC); head turn coefficient of variation (HTCoV); head turn duration (HTD); head turn excursion (HTE); head turn frequency (HTF) and head turn velocity (HTVel).

As depicted in Figure 4, we analyzed the head turn measures in regard to the two defined groups.

For the number of head turns (HTC), we found a significant difference (p<.001) between the groups with a mean of M=9.81 (SD=0.70) for inexperienced compared to experienced players M=8.93 (SD=0.68). Further, the mean variability of velocity (HTCoV) was significantly higher (p<.001) for the more experienced athletes M=99.96 (SD=4.78) % than for their counterparts M=95.14 (SD=2.91) %. For HTD only a small non-significant difference between the two groups could be observed with M=389.15 (SD=17.44) ms and M=385.53 (SD=21.73) ms for inexperienced and experienced players, respectively. Nevertheless, the less experienced group showed a significantly higher (p<.001) HTE with a mean of M=66.72 (SD=4.61) deg, whereas their counterparts had an excursion of M=58.94 (SD=5.02) degrees. Further, we could observe differences in HTF, with a significance of p<.001. Inexperienced and experienced players on average performed M=1.32 (SD=0.06) and M=1.20 (SD=0.08) head turns per second, respectively. In terms of the overall mean yaw-velocity (HTVel), results indicate a significantly (p<.001) higher value of M=122.82 (SD=7.31) ° s^−1^ for the inexperienced group and M=98.67 (SD=7.25) ° s^−1^ for experienced athletes.

### 5.2. Focus of Attention

**Figure 5:**
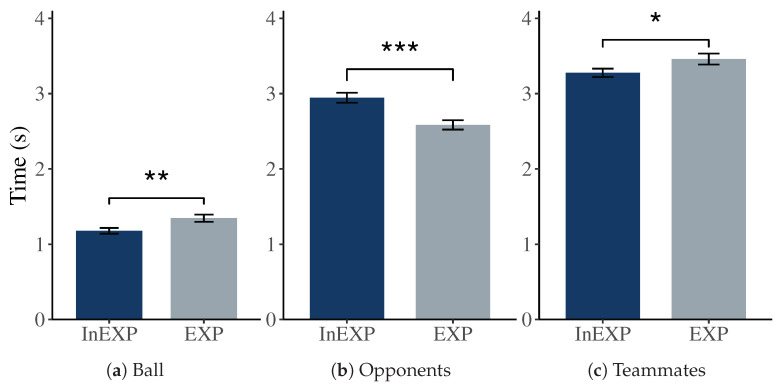
The bar charts depict the mean and standard error of the amounts of time during which inexperienced (InEXP) and experienced (EXP) soccer players focused on the ball (**a**), opponents (**b**) and teammates (**c**). * p<.05; ** p<.01; *** p<.001.

For the FOA, we calculated the duration of how long the three different object types, ball, opponent and teammate, were closest to athletes’ cFOV during each play. A significant difference (p=0.007) for the ball between the two groups could be observed. Experienced players had a prolonged focus on the ball with a mean of M=1.35 (SD=0.28) s, whereas inexperienced athletes only spent M=1.18 (SD=0.23) s looking at the ball (see Figure 5).

For opponents and teammates we also could observe a significant difference between the two groups with p<.001 and p=.049, respectively. Inexperienced players observed the opponents on average for M=2.95 (SD=0.39) s and the teammates for M=3.28 (SD=0.33) s. Contrary, experienced players had, on average, the opponents for M=2.58 (SD=0.37) s and teammates for M=3.46 (SD=0.44) s within their cFOV.

### 5.3. Recognition and Recall

The RRS reflects the performance of recognized marked agents by the participants. The calculated Welch’s two-sample t-test resulted in a significant difference between the two groups of p<.001. Results show that the inexperienced players with correct identification of M=97.67 (SD=2.23) % outperformed their counterparts, which showed a performance of M=93.79 (SD=5.44) %.

### 5.4. Presence and User Experience

Results of the UEQ showed that inexperienced athletes perceived the system to be significantly more stimulating than experienced players. Further, a significant difference between the two groups was found for novelty (NOV). Less experienced players rated this factor higher compared to their counterparts. Table 1 summarises the results of the UEQ.

No significant difference could be found for the results of the presence questionnaire between the two groups. Inexperienced athletes (M=4.70, SD=1.46) rated the sensory factor higher than their counterparts (M=4.25, SD=1.46). The more experienced group (M=4.67, SD=1.26) perceived a higher level of realism than their counterparts (M=4.44, SD=1.54). Comparable results could be shown for distraction with a mean of M=5.52 (SD=1.53) for inexperienced and M=5.54 (SD=1.25) for experienced athletes. Also, for the factor control experienced athletes showed a higher rating (M=5.05, SD=1.33) than the less experienced (M=5.04, SD=1.38).

### 5.5. Correlations

**Figure 6:**
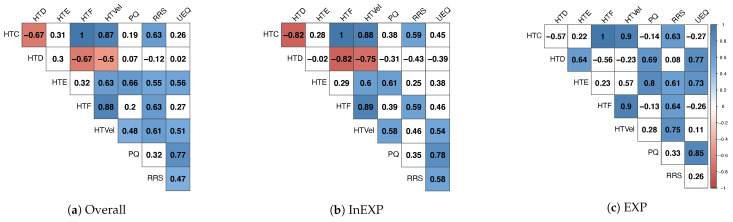
The three different correlation matrices depict the Pearson’s linear correlation coefficients between the head turn characteristics, the RRS and the subjectively perceived presence and user experience of all (**a**), inexperienced (InEXP) (**b**) and experienced (EXP) (**c**) athletes. Significant correlations are marked by color a gradient.

We further investigated the correlations of the individual measures, including the subjectively experienced presence and the user experience of the athletes. Figure 6a depicts the correlation matrix of all athletes regarding the different measures by highlighting the significant results of the pairwise calculated Pearson’s correlation coefficient in color.

Results showed a strong linear positive correlation for the RRS with HTC, HTE, HTF, as well as HTVel, respectively.

Additionally, the presence scores of the participants indicate a significant linear correlation with HTE and HTVel. As depicted in Figure 6b,c, we further investigated the correlations for the individual experience levels. For the less experienced group, we could observe a significantly high correlation for presence with HTE and HTVel, whereas for the experienced group, presence correlated significantly with HTE and HTD.

The results of the UEQ showed a high positive correlation with the PQ as well as a moderate correlation when compared to the RRS. In line with the PQ, significant positive correlations could further be observed with HTE and HTVel. For the inexperienced group the UEQ scores correlated with the RRS and the PQ. In terms of the HTVel a correlation of r=.54,p<.024 could be observed. Comparable to these results, the user experience results of the experienced group showed a significant high correlation with PQ and HTE as well as HTD.

When looking at the individual subcategories of the UEQ and the relevant head turn measures for both groups, HTE significantly correlated with attractiveness and dependability. Comparable HTVel showed a significant correlation with attractiveness and dependability. Further, a moderate significant correlation with perspicuity could be observed. The RRS correlated significantly with the dimensions attractiveness and perspicuity.

The correlation matrices in Figure 6b,c depict the significant results of the individual measures for the inexperienced and experienced group, respectively.

## 6. Discussion

In summary, our results show that the proposed method can identify differences in VEA assessed by head motion characteristics between experienced and less experienced soccer players. We found that the head turn performance, the FOA, and the RRS were explicitly impacted by athletes’ experience level and may therefore also act as discriminator or judgment of a specific athlete’s performance.

### 6.1. Head Turn Performance

Concerning the head turn performance, our study could show that the results of the developed VR-based assessment pipeline are comparable to previously conducted in vivo studies. McGuckian et al. compared the head activity of differently skilled youth players using the Footbonaut, which allows the investigation of the visual search behavior of soccer players, before and during an action [16]. They report that superior soccer players seem to make less head turns and further have a lower excursion of these turns. In our study, a slightly lower frequency of head turns per second could be observed (M=1.28) compared to the in vivo measurements of McGuckian et al. (M=1.75) [16,25]. One reason for this could be that the additional weight of the deployed HMD influenced the behavior of participants. Meaning that a lower amplitude of angular velocity might be observed when wearing the headset, which consequently results in a reduced number of head turns. Future research could determine the actual influence of weight on users’ behavior. Further, the participants may have slightly changed their behavior due to the relatively naive use of novel technology, despite our acclimatization. Based on the findings of future studies investigating the matter, the underlying head turn detection algorithm could be optimized.

Nevertheless, results in this study are comparable to in vivo experiments and suggest that our method can objectively assess VEA of athletes, and therefore optimize the examination of perceptual-cognitive skills in sports.

In our study, experienced player had a significantly lower head turn frequency. This may be caused by a beneficial attitude of orienting their body for action and observation concerning relevant events on the pitch. Consequently, they do not need of adapt their position and orientation that frequently compared to less experienced players [16,61]. According to literature, professional team athletes also show superior skills and knowledge in handling and filtering relevant information during a game. Since it is more demanding for amateurs to decide on decisive visual cues, a more intense exploration of the environment can be assumed [62]. Our findings support this theory by revealing a higher frequency and excursion of head turns for the less experienced group during the study conducted in this work.

Contrary to the absolute amount and frequency of head turns, the duration of head turns did not seem to significantly differ between the two groups. This indicates that even though athletes with less experience show a higher exploratory behavior, this is not influenced by the individual time a movement takes. We could observe an increase in HTD over trials which could be related to the need of habituating to motion in an immersive VR [63]. Highly dynamic soccer plays were used to evaluate the behavior of athletes and to provide a comparable assessment to in vivo situations. Almost all participants had only minor experiences with an HMD and the task to perform in an immersive VE. Although a habituation phase was included in the study design, the need of getting comfortable with the VE might have caused the differences in head turn duration and consequently in excursion and frequency over trials. Even though the experience with and habituation to VR is to be considered, our results could also show that this influence seems to apply in the same way to both groups.

Another interesting observation is the difference in the variability of velocity between the two groups (HTCoV). More experienced players showed a significantly higher volatility. Meaning, the velocity characteristic of less experienced athletes can be described as performing more frequent spontaneous movements in comparison to their counterparts, who tend to have a higher standard deviation, but a lower average angular velocity. To the best of our knowledge, this effect has not been previously reported. Future research should further investigate this finding using in vivo soccer scenarios.

### 6.2. Focus of Attention

Our results regarding the FOA show that low experienced players tend to focus significantly longer on opponents compared to more experienced ones. Further, the latter had a significantly higher interest in observing the ball and spent more time focusing on teammates. These findings go in line with previous in vivo experiments [12,41,64]. For example, Vaeyens et al. examined the decision-making and search behavior of soccer players. They used realistic film simulations of offensive plays in combination with eye tracking assessments. Results suggest that higher-skilled players tend to fixate more extensively on the player in ball possession compared to less skilled players [12]. An explanation could be the advanced skill of gathering and selecting peripheral information of professional players. It gives them the possibility to have a central focus of the action, but receive relevant context information at the same time. Unlike, amateur players tend to shift their focus more towards the information sources to be explored [32]. During our experiment, the more experienced players spent more time focusing on the ball and the teammates, which indicates that they showed higher interest in the ball position and the player in ball possession. Our method did not explicitly investigate the player in ball possession, but rather the focus on the ball, opponents, and teammates individually. Future research could include a more detailed differentiation of objects of interest (e.g., including the player in ball possession).

Recently published work by Aksum et al. indicates that the search behavior of professional athletes differs between laboratory results [37,65] and actual on-pitch performance. They conducted a study where Norwegian premier league midfielders’ gaze behavior was analyzed during an 11 vs. 11 play. Precisely, they observed that in competitive situations, professional players’ fixation locations were mainly on the ball, opponent, and teammate category. Since their observations are partially contradictive with previous literature, they conclude that traditional laboratory settings may not be sufficient to provoke the behavior of soccer players performed in dynamic soccer competitions [41]. The results found in this VR study seem to agree with the observations of Aksum et al. [41].

Overall, we provided a new approach to determine athletes’ FOA in dynamic VR-based soccer scenarios. Its capability to distinguish different experience levels contributes by creating a more holistic view of athletes’ behavioral characteristics in dynamic game situations and hence informs future research and development. To enable a more detailed analysis for future research in VR, this approach could be optimized to allow the differentiation of more object types like the player in possession of the ball. Another alternative would be the investigation of gaze behavior using eye tracking to be able to not only provide information of the center focus area of an athlete, but additionally determine the area of interest. However, results of exploratory behavior of athletes seem not to be clear when looking at previous literature [12,37,64,65].

Nevertheless, the applied method could provide comparable results to previous in vivo experiments. This indicates that the employed VR-based approach is an effective alternative to in vivo measurements for assessing soccer players’ FOA. Further, having a holistic view of the athlete by providing more head-based exploratory measures can be beneficial to understand the behavior of athletes in dynamic competitive situations.

### 6.3. Recognition and Recall

In our study, the findings for the RRS showed that, unexpectedly, less experienced players reached a significantly higher score of approximately 97% correct answers, whereas their more experienced counterparts reached an average score of 94%. However, previous literature indicates that experienced players only possess superior cognitive knowledge and processing when it comes to structured plays [66]. The reason for this is that experienced soccer players have developed knowledge that facilitates meaningful associations between players’ positions and their relevance in game-specific situations [66,67]. The marking of the player agents in this study was rather unstructured in a way that the number of marked players varied over plays. The non-task specific (i.e., unstructured) nature of these visual hints might be one reason for the higher recall error of experienced players observed in this study.

Another reason for this finding could be the restriction in peripheral vision. According to previous in vivo studies, superior soccer players make more use of peripheral information than their counterparts [11,65]. Since the utilized HMD provides a FOV of 110°, the peripheral vision of the athletes was restricted during the experiment and could therefore have impacted their recall performance.

In addition, we found that the RRS strongly correlated with the HTC, HTE, HTF, and HTVel. These results revealed that a more intensified overall head activity led to a higher recognition and recall ability for unstructured visual information during the experiment. While for less experienced players this could be observed for the frequency of head turns, experienced players’ RRS also showed a strong positive correlation for HTE. Previous literature states that extensive exploratory activity within the period before receiving the ball of soccer players results in more successful actions with the ball [43]. In this regard, our results provide supporting evidence.

In summary, the assessed RRS could show significant differences between the two examined experience levels. The positive correlations found between the head activity measures applied in this work and the RRS underpin the assumption that higher exploratory activity in team sports leads to more information retrieval decisive for successful decision-making [42]. However, in our experiment, less experienced players outperformed their counterparts in recalling the number of displayed stimuli, which could result from the unstructured nature of the applied task. To get deeper insights into the applicability of realistic and dynamic VR environments for the evaluation of athletes’ recall and recognition behavior further studies using a structured task design should be conducted.

### 6.4. Presence and User Experience

The employed VR simulation achieved a high score when comparing user experience results to the UEQ benchmark dataset, except for the factor dependability. The reason for the low rating might be that the task of the participants within this study was to only explore the environment and hence no direct interaction possibilities to manipulate the VE were given. For future research, this could be improved by providing intuitive interaction in such a sports scenario as foot interaction.

For the UEQ we could observe significant differences for the factors novelty and stimulation between the two investigated experience levels. Reasons for such a difference may lie in the fact that higher experienced athletes are more used to technique supported assessment and training methods. Prior use of HMDs should not have influenced the results, since the experience with those devices was comparable between the two groups. Previous literature could show that athletes’ personality traits affect the perceived user experience in VEs [10]. Hence, the player types and also experience levels seem to be relevant when it comes to decisive motivation factors like novelty and stimulation [68]. We therefore follow the suggestion of Wirth et al. to not only consider task-relevant aspects (e.g., realistic dynamic sports scenes), but also consider hedonic quality (i.e., novelty and stimulation) as criteria when designing VR-based assessment and training simulations in sports.

We could not observe significant differences in presence between the two groups. Concerning the UEQ, this is an interesting finding, since even though the experience level seems to significantly influence the perception of a simulation’s hedonic quality, the same does not apply for the perceived presence. From our perspective, this underpins the suggestion of having additional quality measures besides the factor of presence to get a holistic view of the user experience when evaluating VR simulations.

The subjectively perceived presence of athletes showed a significant positive correlation with HTE and HTVel. While this could be observed for less experienced players, presence correlated significantly with HTE and HTD for more experienced athletes. From our perspective, HTE could be a relevant parameter, to objectively define presence. As stated by Usoh et al., an increased presence results in a greater similarity of comparable users’ real-world behavior in VEs. Therefore, such measures yield great potential to relate to presence [69]. This potential could, among other research [70,71], be shown by Freeman et al. [72]. Their study of presence revealed effects on postural response when users perceived a variety of stimuli in an immersive VE [72]. Moreover, behavior measures like posture seem to be efficient since users are not conscious of their observation and hence subjective biases can be excluded [71].

However, to the best of our knowledge, there is no research work yet which could show that users’ excursion of head turns (HTE) strongly correlates with their subjectively perceived presence for an exploration task in VR.

## 7. Conclusions

In summary, this work contributes in various ways. We created a new analysis pipeline consisting of multiple head motion characteristics that have not been investigated in a VE before. These measures were applied within a dynamic recognition and recall task to examine the VEA of inexperienced and experienced soccer players. Further, the experienced presence and overall user experience of participants regarding the VE was evaluated. Our results show that our approach could be successfully used to distinguish between different experience levels of players and can therefore be used for VEA assessment. In team sports, this is highly relevant since it enables to design more efficient assessment processes and hence is supportive in creating a more holistic view of athletes to optimize individual performance improvement processes.

We further found that the subjective measure of presence strongly correlated with the HTE of participants. Since to the best of our knowledge, the correlation between those two variables has not been examined in any previous research, we see this as an additional major contribution of this work, that needs to be further investigated.

Results of the head motion characteristics showed a significant difference between the two investigated groups in terms of HTC, HTCoV, HTE, HTF and HTVel. The findings go in line with comparable in vivo studies conducted in team sports. Consequently, this makes these measures a promising tool for the assessment and training of athletes in VR. The results further strengthen the applicability of VR as an efficient immersive medium for performance analysis in sports.

Another contribution of this work is the exploration of athletes’ focus of attention in VR. Therefore, an approach was chosen and evaluated that measured the focus time of soccer players on the visual ball, opponents and teammates. Significant differences were found for the two examined groups varying in experience level. This could be further improved in the future by using eye tracking.

Less experienced athletes outperformed their counterparts in the recognition and recall task. One reason for this might be the lack of full natural peripheral vision caused by the used HMD. Professional soccer players tend to derive decisive information from this visual area more extensively than amateurs [11]. This is currently a limiting factor of the hardware devices, therefore, it can be expected to vanish with newer generations of HMDs.

For the subjective experience of athletes, we found significant differences between the two groups for hedonic quality (i.e., stimulation and novelty), whereas no significant variation could be observed for presence. However, in sports, motivation and the experienced stimulation of the athlete play a decisive role. Therefore, to get a holistic view on the user experience for the VE to be developed, derived from the observations made in the conducted study we recommend to not only investigate the presence of an athlete, but also consider motivational linked factors like novelty and/or stimulation.

## Figures and Tables

**Table 1:** Results of the user experience questionnaire.

Subcat.	InEXP (±*SEM*) ^†^	EXP (±*SEM*) ^†^	*p*	*BM_UEQ_*
ATT	5.89(±0.23)	5.32(±0.22)		good
DEP	5.26(±0.15)	4.94(±0.17)		below average
EFF	5.35(±0.17)	5.08(±0.16)		above average
NOV	5.81(±0.19)	5.21(±0.20)	*	excellent
PER	5.93(±0.23)	5.81(±0.21)		good
STI	6.04(±0.20)	5.21(±0.25)	*	excellent

Note. ^†^ Mean (±standard error of the mean); * *p* < .05; *BM_UEQ_*: UEQ benchmark categories [60]; ATT: Attractiveness; DEP: Dependability; EFF: Efficiency; NOV: Novelty; PER: Perspicuity; STI: Stimulation; InEXP: Inexperienced; EXP: Experienced.

## Abbreviations

The following abbreviations are used in this manuscript:

2D	two-dimensional
3D	three-dimensional
cFOV	center field of view
FOA	focus of attention
FOR	field of regard
FOV	field of view
HMD	head-mounted display
HTC	head turn count
HTCoV	head turn coefficient of variation
HTD	head turn duration
HTE	head turn excursion
HTF	head turn frequency
HTVel	head turn velocity
IMU	Inertial Measurement Unit
LPS	local positioning system
MEMS	Micro-Electro-Mechanical Systems
PCS	perceptual-cognitive skills
PQ	presence questionnaire
RRS	recognition and recall score
UEQ	user experience questionnaire
UWB	ultra-wideband
VEA	visual exploratory activity
VE	virtual environment
VR	virtual reality

## References

[1] Andreassen K., Johansen D., Johansen H., Baptista I., Pettersen S.A., Riegler M., Halvorsen P. Real-time Analysis of Physical Performance Parameters in Elite Soccer. Proceedings of the 2019 International Conference on Content-Based Multimedia Indexing (CBMI).

[2] Redwood-Brown A., Cranton W., Sunderland C. (2012). Validation of a real-time video analysis system for soccer. Int. J. Sports Med..

[3] Philippaerts R.M., Vaeyens R., Janssens M., Van Renterghem B., Matthys D., Craen R., Bourgois J., Vrijens J., Beunen G., Malina R.M. (2006). The relationship between peak height velocity and physical performance in youth soccer players. J. Sports Sci..

[4] Jovanovic M., Sporis G., Omrcen D., Fiorentini F. (2011). Effects of speed, agility, quickness training method on power performance in elite soccer players. J. Strength Cond. Res..

[5] Kaplan T., Erkmen N., Taskin H. (2009). The evaluation of the running speed and agility performance in professional and amateur soccer players. J. Strength Cond. Res..

[6] Sheppard J., Young W.B., Doyle T., Sheppard T., Newton R.U. (2006). An evaluation of a new test of reactive agility and its relationship to sprint speed and change of direction speed. J. Sci. Med. Sport.

[7] Williams A.M., Reilly T. (2000). Talent identification and development in soccer. J. Sports Sci..

[8] Abernethy A., Cote J., Baker J. Learning to be an expert: Factors underpinning the development of exceptional decision-making skills in athletes. Proceedings of the 2000 Pre-Olympic Congress: International Congress on Sport Science, Sports Medicine and Physical Education.

[9] Helsen W.F., Starkes J.L. (1999). A multidimensional approach to skilled perception and performance in sport. Appl. Cogn. Psychol. Off. J. Soc. Appl. Res. Mem. Cogn..

[10] Wirth M., Gradl S., Mehringer W.A., Kulpa R., Rupprecht H., Poimann D., Laudanski A.F., Eskofier B.M. Assessing Personality Traits of Team Athletes in Virtual Reality. Proceedings of the 2020 IEEE Conference on Virtual Reality and 3D User Interfaces Abstracts and Workshops (VRW).

[11] Mann D.T., Williams A.M., Ward P., Janelle C.M. (2007). Perceptual-cognitive expertise in sport: A meta-analysis. J. Sport Exerc. Psychol..

[12] Vaeyens R., Lenoir M., Williams A.M., Mazyn L., Philippaerts R.M. (2007). The effects of task constraints on visual search behavior and decision-making skill in youth soccer players. J. Sport Exerc. Psychol..

[13] Williams A.M., Davids K., Williams J.G.P. (1999). Visual Perception and Action in Sport.

[14] McGuckian T.B., Cole M.H., Pepping G.J. (2018). A systematic review of the technology-based assessment of visual perception and exploration behaviour in association football. J. Sports Sci..

[15] Kredel R., Vater C., Klostermann A., Hossner E.J. (2017). Eye-tracking technology and the dynamics of natural gaze behavior in sports: A systematic review of 40 years of research. Front. Psychol..

[16] McGuckian T.B., Beavan A., Mayer J., Chalkley D., Pepping G.J. (2020). The association between visual exploration and passing performance in high-level U13 and U23 football players. Sci. Med. Footb..

[17] Ali A. (2011). Measuring soccer skill performance: A review. Scand. J. Med. Sci. Sports.

[18] Buchheit M., Simpson M., Al Haddad H., Bourdon P., Mendez-Villanueva A. (2012). Monitoring changes in physical performance with heart rate measures in young soccer players. Eur. J. Appl. Physiol..

[19] Wirth M., Gradl S., Poimann D., Schaefke H., Matlok J., Koerger H., Eskofier B.M. (2018). Assessment of Perceptual-Cognitive Abilities among Athletes in Virtual Environments: Exploring Interaction Concepts for Soccer Players. Proceedings of the 2018 Designing Interactive Systems Conference (DIS ’18).

[20] Zrenner M., Gradl S., Jensen U., Ullrich M., Eskofier B.M. (2018). Comparison of different algorithms for calculating velocity and stride length in running using inertial measurement units. Sensors.

[21] Bowman D.A., McMahan R.P. (2007). Virtual reality: How much immersion is enough?. Computer.

[22] Bailenson J., Patel K., Nielsen A., Bajscy R., Jung S.H., Kurillo G. (2008). The effect of interactivity on learning physical actions in virtual reality. Media Psychol..

[23] Huang Y., Churches L., Reilly B. A case study on virtual reality American football training. Proceedings of the 2015 Virtual Reality International Conference.

[24] McGuckian T.B. (2016). A wearable inertial sensor for improved measurement of exploration behaviour in sport compared to notational analysis. J. Fit. Res..

[25] McGuckian T.B., Cole M.H., Chalkley D., Jordet G., Pepping G.J. (2019). Visual exploration when surrounded by affordances: Frequency of head movements is predictive of response speed. Ecol. Psychol..

[26] Gradl S., Eskofier B.M., Eskofier D., Mutschler C., Otto S. (2016). Virtual and Augmented Reality in Sports—An Overview and Acceptance Study. Proceedings of the 2016 ACM International Joint Conference on Pervasive and Ubiquitous Computing Adjunct—UbiComp ’16.

[27] Rojas Ferrer C.D., Shishido H., Kitahara I., Kameda Y. (2020). Read-the-game: System for skill-based visual exploratory activity assessment with a full body virtual reality soccer simulation. PLoS ONE.

[28] Wood G., Wright D., Harris D., Pal A., Franklin Z., Vine S. (2021). Testing the construct validity of a soccer-specific virtual reality simulator using novice, academy, and professional soccer players. Virtual Real..

[29] Cutting J.E. (1986). Perception with an Eye for Motion.

[30] Haber R.N., Hershenson M. (1973). The Psychology of Visual Perception.

[31] McMorris T. (2014). Acquisition and Performance of Sports Skills.

[32] Roca A., Ford P.R., McRobert A.P., Williams A.M. (2011). Identifying the processes underpinning anticipation and decision-making in a dynamic time-constrained task. Cogn. Process..

[33] Dicks M., Button C., Davids K., Chow J.Y., Van der Kamp J. (2017). Keeping an eye on noisy movements: On different approaches to perceptual-motor skill research and training. Sports Med..

[34] Jordet G. (2004). Perceptual Expertise in Dynamic and Complex Competitive Team Contexts: An Investigation of Elite Football Midfield Players. Ph.D. Thesis.

[35] Ward P., Williams A.M. (2003). Perceptual and cognitive skill development in soccer: The multidimensional nature of expert performance. J. Sport Exerc. Psychol..

[36] Williams A.M. (2000). Perceptual skill in soccer: Implications for talent identification and development. J. Sports Sci..

[37] Williams A.M., Davids K., Burwitz L., Williams J.G. (1994). Visual search strategies in experienced and inexperienced soccer players. Res. Q. Exerc. Sport.

[38] Roca A., Ford P.R., McRobert A.P., Williams A.M. (2013). Perceptual-cognitive skills and their interaction as a function of task constraints in soccer. J. Sport Exerc. Psychol..

[39] Cañal-Bruland R., Lotz S., Hagemann N., Schorer J., Strauss B. (2011). Visual span and change detection in soccer: An expertise study. J. Cogn. Psychol..

[40] Woolley T., Crowther R., Doma K., Connor J. (2015). The use of spatial manipulation to examine goalkeepers’ anticipation. J. Sports Sci..

[41] Aksum K.M., Magnaguagno L., Bjørndal C.T., Jordet G. (2020). What do football players look at? An eye-tracking analysis of the visual fixations of players in 11 v 11 elite football match play. Front. Psychol..

[42] Eldridge D., Pulling C., Robins M.T. (2013). Visual exploratory activity and resultant behavioural analysis of youth midfield soccer players. J. Hum. Sport Exerc..

[43] Jordet G., Bloomfield J., Heijmerikx J. The hidden foundation of field vision in English Premier League (EPL) soccer players. Proceedings of the MIT Sloan Sports Analytics Conference.

[44] Jordet G., Pepping G.J., Cappuccio M., Press M. (2019). Flipping sport psychology theory into practice. A context-and behavior centered approach. Handbook of Embodied Cognition and Sport Psychology.

[45] Chalkley D., Shepherd J.B., McGuckian T.B., Pepping G.J. (2018). Development and Validation of a Sensor-Based Algorithm for Detecting the Visual Exploratory Actions. IEEE Sens. Lett..

[46] McGuckian T.B., Cole M.H., Jordet G., Chalkley D., Pepping G.J. (2018). Don’t turn blind! The relationship between exploration before ball possession and on-ball performance in association football. Front. Psychol..

[47] McGuckian T.B., Cole M.H., Chalkley D., Jordet G., Pepping G.J. (2020). Constraints on visual exploration of youth football players during 11v11 match-play: The influence of playing role, pitch position and phase of play. J. Sports Sci..

[48] Romeas T., Chaumillon R., Labbé D., Faubert J. (2019). Combining 3D-MOT with sport decision-making for perceptual-cognitive training in virtual reality. Percept. Mot. Ski..

[49] Doi M., Takai T., Chihara K. (2000). VR American football simulator with cylindrical screen. International Conference on Virtual Worlds.

[50] McLeod P., Reed N., Gilson S., Glennerster A. (2008). How soccer players head the ball: A test of optic acceleration cancellation theory with virtual reality. Vis. Res..

[51] Hoinville T., Naceri A., Ortiz J., Bernier E., Chellali R. Performances of experienced and novice sportball players in heading virtual spinning soccer balls. Proceedings of the 2011 IEEE Virtual Reality Conference.

[52] Dessing J.C., Craig C.M. (2010). Bending it like Beckham: How to visually fool the goalkeeper. PLoS ONE.

[53] Bideau B., Kulpa R., Vignais N., Brault S., Multon F., Craig C. (2009). Using virtual reality to analyze sports performance. IEEE Comput. Graph. Appl..

[54] Ferrer C.D.R., Kitahara I., Kameda Y. Read-the-game skill evaluation by analyzing head orientation in immersive VR. Proceedings of the 2017 3DTV Conference: The True Vision-Capture, Transmission and Display of 3D Video (3DTV-CON).

[55] Bjurwill C. (1993). Read and react: The football formula. Percept. Mot. Ski..

[56] IFAB (2020). Laws of the Game 2020/21. https://www.theifab.com/laws.

[57] Schafer R.W. (2011). What is a Savitzky-Golay filter? [lecture notes]. IEEE Signal Process. Mag..

[58] Witmer B.G., Singer M.J. (1998). Measuring presence in virtual environments: A presence questionnaire. Presence.

[59] Laugwitz B., Held T., Schrepp M. (2008). Construction and evaluation of a user experience questionnaire. Symposium of the Austrian HCI and Usability Engineering Group.

[60] Schrepp M., Hinderks A., Thomaschewski J. (2017). Construction of a Benchmark for the User Experience Questionnaire (UEQ). IJIMAI.

[61] Oppici L., Panchuk D., Serpiello F.R., Farrow D. (2017). Long-term practice with domain-specific task constraints influences perceptual skills. Front. Psychol..

[62] Starkes J.L., Ericsson K.A. (2003). Expert Performance in Sports: Advances in Research on Sport Expertise.

[63] Howarth P.A., Hodder S.G. (2008). Characteristics of habituation to motion in a virtual environment. Displays.

[64] Vaeyens R., Lenoir M., Williams A., Mazyn L., Philippaerts R. (2007). Visual search behavior and decision-making skill in soccer. J. Mot. Behav..

[65] Williams A., Davids K. (1998). Visual search strategy, selective attention, and expertise in soccer. Res. Q. Exerc. Sport.

[66] Williams M., Davids K., Burwitz L., Williams J. (1993). Cognitive knowledge and soccer performance. Percept. Mot. Ski..

[67] Garland D.J., Barry J.R. (1991). Cognitive advantage in sport: The nature of perceptual structures. Am. J. Psychol..

[68] Berlyne D.E. (1950). Novelty and curiosity as determinants of exploratory behaviour. Br. J. Psychol..

[69] Usoh M., Alberto C., Slater M. (1996). Presence: Experiments in the Psychology of Virtual Environments.

[70] Cooper N., Milella F., Pinto C., Cant I., White M., Meyer G. (2018). The effects of substitute multisensory feedback on task performance and the sense of presence in a virtual reality environment. PLoS ONE.

[71] Coelho C., Tichon J., Hine T.J., Wallis G., Riva G. (2006). Media presence and inner presence: The sense of presence in virtual reality technologies. From Communication to Presence: Cognition, Emotions and Culture Towards the Ultimate Communicative Experience.

[72] Freeman J., Avons S.E., Meddis R., Pearson D.E., IJsselsteijn W. (2000). Using behavioral realism to estimate presence: A study of the utility of postural responses to motion stimuli. Presence Teleoperators Virtual Environ..

